# Novel genotypes of *Coxiella burnetii* circulating in rats in Yunnan Province, China

**DOI:** 10.1186/s12917-022-03310-8

**Published:** 2022-05-27

**Authors:** Mengjiao Fu, Peisheng He, Xuan OuYang, Yonghui Yu, Bohai Wen, Dongsheng Zhou, Xiaolu Xiong, Qinghong Yuan, Jun Jiao

**Affiliations:** 1 State Key Laboratory of Pathogen and Biosecurity, Beijing Institute of Microbiology and Epidemiology, Beijing, People’s Republic of China; 2 Department of Epidemiology and Biostatistics, School of Public Health, Anhui Medical University, Hefei, People’s Republic of China; 3 Yunnan Institute of Endemic Diseases Control and Prevention, Yunnan Provincial Key Laboratory of Natural Focal Disease Control and Prevention, Yunnan, People’s Republic of China

**Keywords:** *Rattus flavipectus*, *Coxiella burnetii*, Genotype, MST, MLVA, China

## Abstract

**Background:**

*Coxiella burnetii* (Cb) is the causative agent of the zoonotic disease Q fever which is distributed worldwide. Molecular typing of Cb strains is essential to find out the infectious source and prevent Q fever outbreaks, but there has been a lack of typing data for Cb strains in China. The aim of this study was to investigate the genotypes of Cb strains in wild rats in Yunnan Province, China.

**Results:**

Eighty-six wild rats (*Rattus flavipectus*) were collected in Yunnan Province and 8 of the 86 liver samples from the wild rats were positive in Cb-specific quantitative PCR (qPCR). The Cb strains from the 8 rats were then typed into 3 genotypes using 10-spacer multispacer sequence typing (MST), and 2 of the 3 genotypes were recognized as novel ones. Moreover, the Cb strains in the wild rats were all identified as genotype 1 using 6-loci multilocus variable number of tandem repeat analysis (MLVA).

**Conclusions:**

This is the first report of genotypic diversity of Cb strains from wild rats in China. Further studies are needed to explore the presence of more genotypes and to associate the genotypes circulating in the wildlife-livestock interaction with those causing human disease to further expand on the epidemiological aspects of the pathogen.

## Background

*Coxiella burnetii* (Cb), an intracellular Gram-negative bacterium, is the causative agent of Q fever, a disease distributed worldwide except in New Zealand [1]. Cb is mainly found in livestock, such as goats, sheep and cattle, where chronic infection is associated with late abortion, stillbirth, and weak offspring [2]. Other reservoirs of Cb include birds, wild rodents and arthropods [3, 4]. Although ticks can transmit Cb in experimental systems, direct transmission of Cb to humans through ticks has never been properly documented [5, 6].

*C. burnetii* infection exhibits various acute and chronic clinical manifestations in humans. Acute Q fever typically presents as a flu-like illness with a high fever, headache, myalgia and malaise, and can develop into chronic Q fever which presents as endocarditis, hepatitis, and/or osteomyelitis [7, 8]. Human acute Cb infections often occur after inhalation of Cb-contaminated aerosols from infected animals, thus occupations that require close contact with livestock have a higher risk of acquiring Q fever [7, 9, 10].

The importance of Q fever has been increasing since the outbreak in the Netherlands from 2007 to 2010, where more than 4000 people became ill [11]. To prevent infection in humans, it is critical to determine the source of infection for each case including patients with Q fever exposed to wildlife. For this purpose, several genotyping methods have been described including sequencing analysis of individual genes like 16S, 23S [12], plasmid-based typing [13, 14], restriction fragment length polymorphism and pulsed-field gel electrophoresis (RFLP-PFGE) analysis [15], single nucleotide polymorphism (SNP) [16], multispacer sequence typing (MST) [17, 18], and multilocus variable number tandem repeats analysis (MLVA) [19]. Of the mentioned methods, MST and MLVA can be applied directly to DNA samples without previous cultivation of the strain, and have proven to be reliable and reproducible.

In China, Cb has been detected in wildlife, but few MST or MLVA genotypes of Cb strains have been reported [20, 21]. This study aimed to genotype Cb strains detected in wild rats in Yunnan Province. This study is the first report of MLVA and MST genotypes of Cb strains in wild rats in China, which provides further insight into the epidemiology and evolution of Q fever.

## Results

### Rodent species identification

**Fig. 1:**
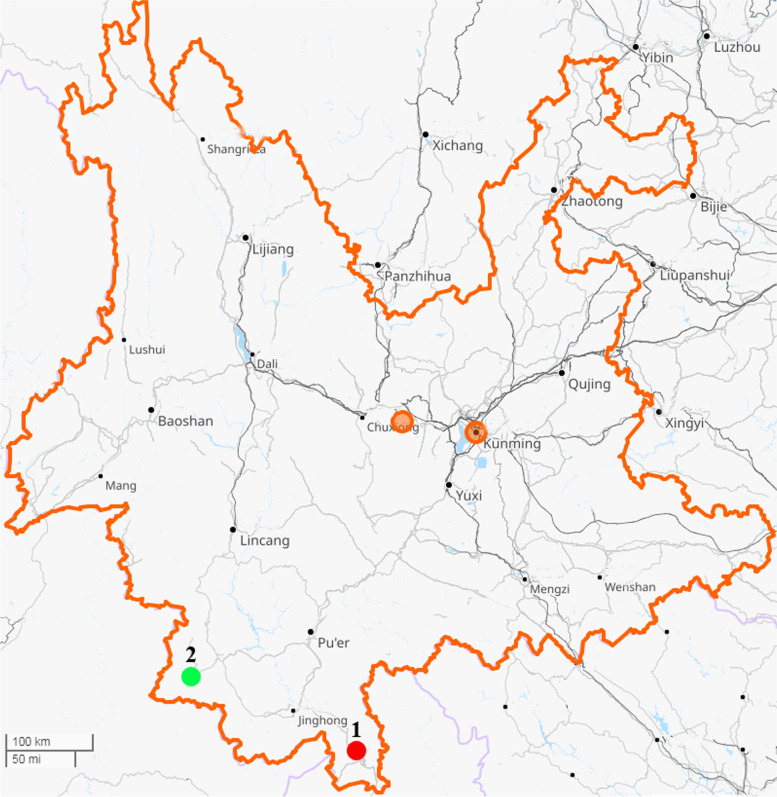
Map of the sampling sites in Yunnan Province, China. Rats were collected in Mengla County (sampling site 1) and Menglian County (sampling site 2) in Yunnan Province

A total of 86 wild rats were collected from wild fields in Mengla County (*n* = 33) and Menglian County (*n* = 53) in Yunnan Province in August 2020 (Fig. 1). All rats were identified as *Rattus flavipectus* based on morphological identifications and confirmed by species-specific PCR and sequencing assays.

### *C. burnetii* detection

The presence of *C. burnetii* in the wild rats was first tested on their liver samples by nested PCR. Eight of 53 liver samples (15.09%, 8/53) collected in Menglian County were positive in PCR targeting Cb *16S rRNA*, and the sequences obtained showed 99.17–99.44% nucleotide sequence identity to those of the known Cb strains in *16S rRNA* comparison. No rats collected in Mengla County were positively detected in PCR.

The liver samples positively detected by Cb-specific PCR were further analyzed by qPCR to assess the Cb loads in the wild rats. As a result, all 8 liver samples showed a Cb concentration ranging from 4.40 × 10^4^ to 2.53 × 10^6^ copies/gram (Table 1).

**Table 1:** Summary of qPCR of the rat samples used in MST and MLVA genotyping

Sample ID	Host	Sample source	Ct in qPCR	Quantification in qPCR (Copies/gram)
4	*Rattus flavipectus*	liver	34.15	1.45 × 10^5^
5	*Rattus flavipectus*	liver	34.52	1.18 × 10^5^
12	*Rattus flavipectus*	liver	34.35	1.34 × 10^5^
16	*Rattus flavipectus*	liver	33.73	1.85 × 10^5^
20	*Rattus flavipectus*	liver	34.28	1.35 × 10^5^
21	*Rattus flavipectus*	liver	36.21	4.40 × 10^4^
22	*Rattus flavipectus*	liver	35.72	5.85 × 10^4^
44	*Rattus flavipectus*	liver	29.07	2.53 × 10^6^

### MST genotyping

Seven of the 8 liver samples positive for Cb were successfully amplified for all 10 spacers in MST genotyping. For samples 4 and 44, the allele codes found in the present study for loci Cox2-Cox5-Cox18-Cox20-Cox22-Cox37-Cox51-Cox56-Cox57-Cox61 were 3–8-5–3-4–1-6–7-6–5 and belonged to MST genotype 16. For sample 12, Cox61 was unable to be sequenced with good resolution, possibly due to poor quantity of DNA.

**Fig. 2:**
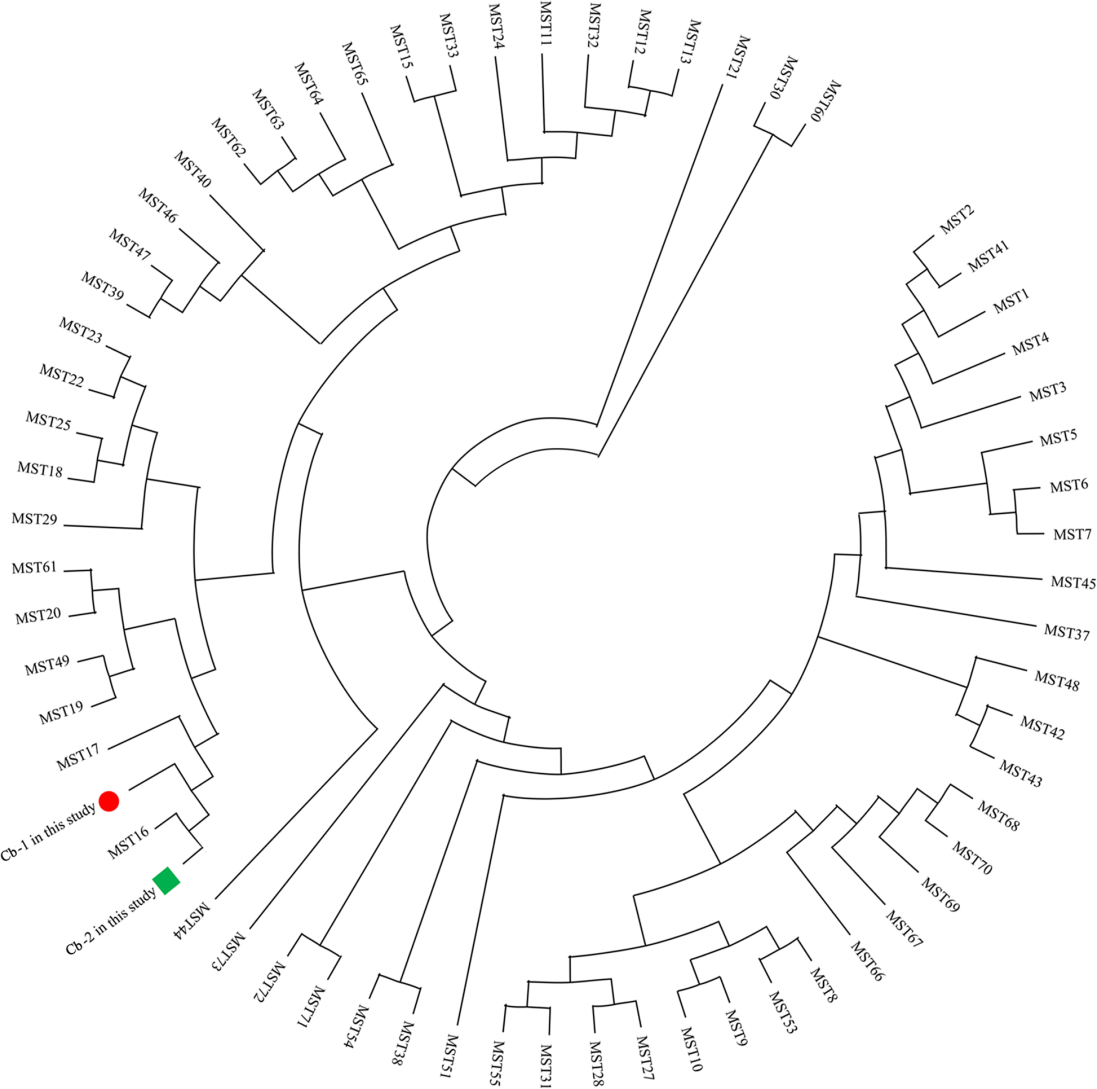
Phylogenetic tree of *Coxiella burnetii* MST genotypes from this study with known genotypes. The obtained MST genotypes identified in this study and data of known MST genotypes based on the MST database (https://ifr48.timone.univ-mrs.fr/mst/coxiella_burnetii/) were used. Sequences were aligned using the MEGA X (Version 10.2.5) software package. Phylogenetic analysis was performed using the unweighted pair group method with arithmetic mean (UPGMA) method. Cb-1: genotype of samples 5, 16, 20, 21; Cb-2: genotype of sample 22

For samples 5, 16, 20, 21 and 22, spacer sequences of Cox 20 and/or Cox37 were diverse from those described in the MST database (Table 2). For Cox 20, mutations T/A at position 504 and G/A at position 513 of allele 3 were present. For Cox 37, a deletion of A at position 42 and a mutation T/G at position 33 of allele 1 were present. These data suggested the presence of new alleles, implying the existence of novel MST genotypes. A phylogenetic tree placed the MST genotypes of these strains (samples 5, 16, 20, 21 and 22) close to MST genotype 16 (Fig. 2).

**Table 2:** MST genotypes of *C. burnetii* detected in rats in the present study

	**MST Loci**										
Sample ID	Cox 2	Cox 5	Cox 18	Cox 20	Cox 22	Cox 37	Cox 51	Cox 56	Cox 57	Cox 61	MST type
4	3	8	5	3	4	1	6	7	6	5	16
5	3	8	5	3	4	Novel	6	7	6	5	Novel
12	3	8	5	3	4	1	6	7	6	N/A	-
16	3	8	5	3	4	Novel	6	7	6	5	Novel
20	3	8	5	3	4	Novel	6	7	6	5	Novel
21	3	8	5	3	4	Novel	6	7	6	5	Novel
22	3	8	5	Novel	4	Novel	6	7	6	5	Novel
44	3	8	5	3	4	1	6	7	6	5	16

*N/A* Negative assembly

### MLVA genotyping

**Fig. 3:**
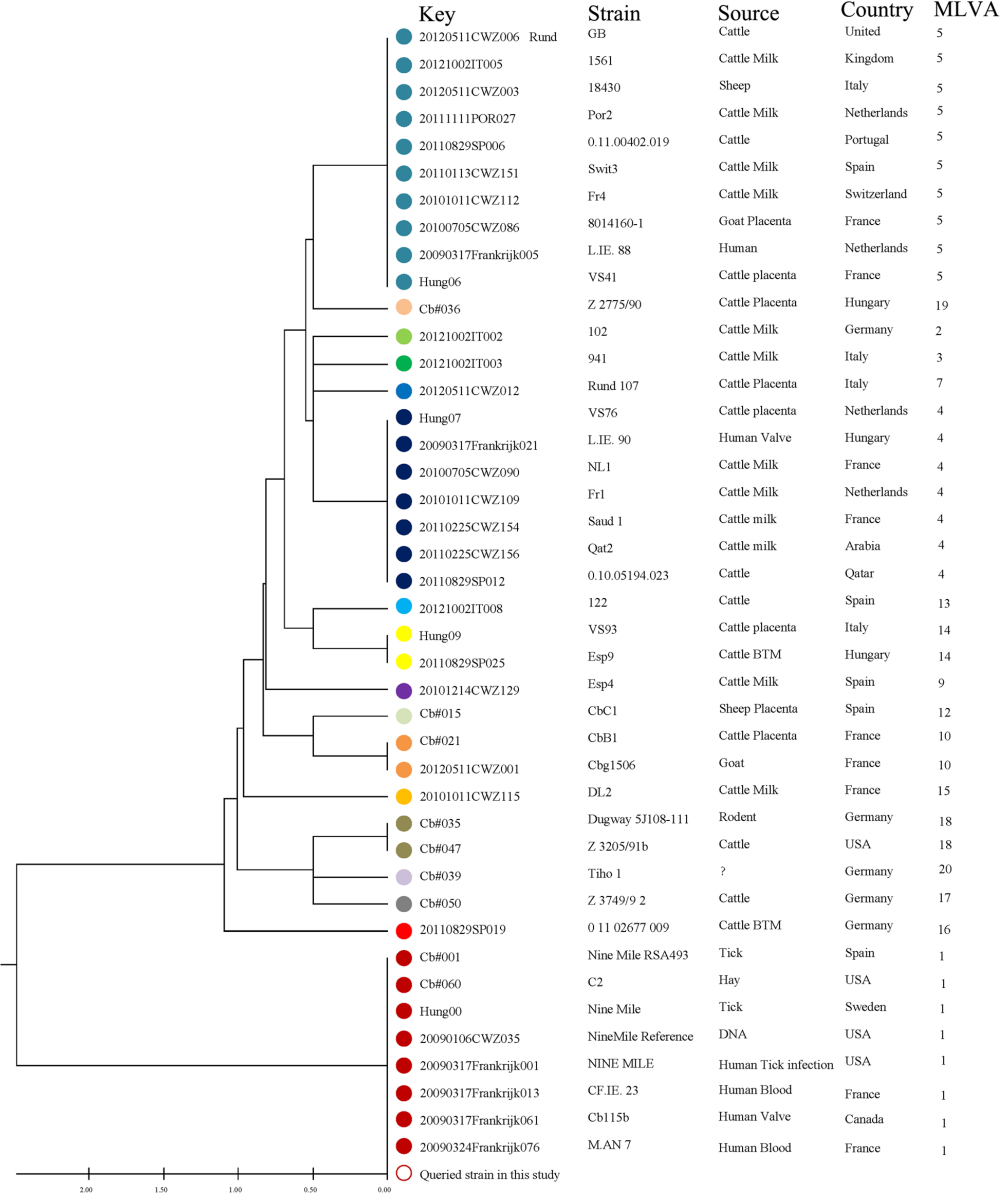
UPGMA cluster analysis of *Coxiella burnetii* MLVA-6 genotypes. All data of selected samples are available in the MLVA-6 database (http://mlva.i2bc.paris-saclay.fr/mlvav4/genotyping/). Strain, source, geographical origin, and MLVA-6 type are indicated. The same genotype was coded with the same color, and the hollow dot indicates the genotype obtained in the present study

**Fig. 4:**
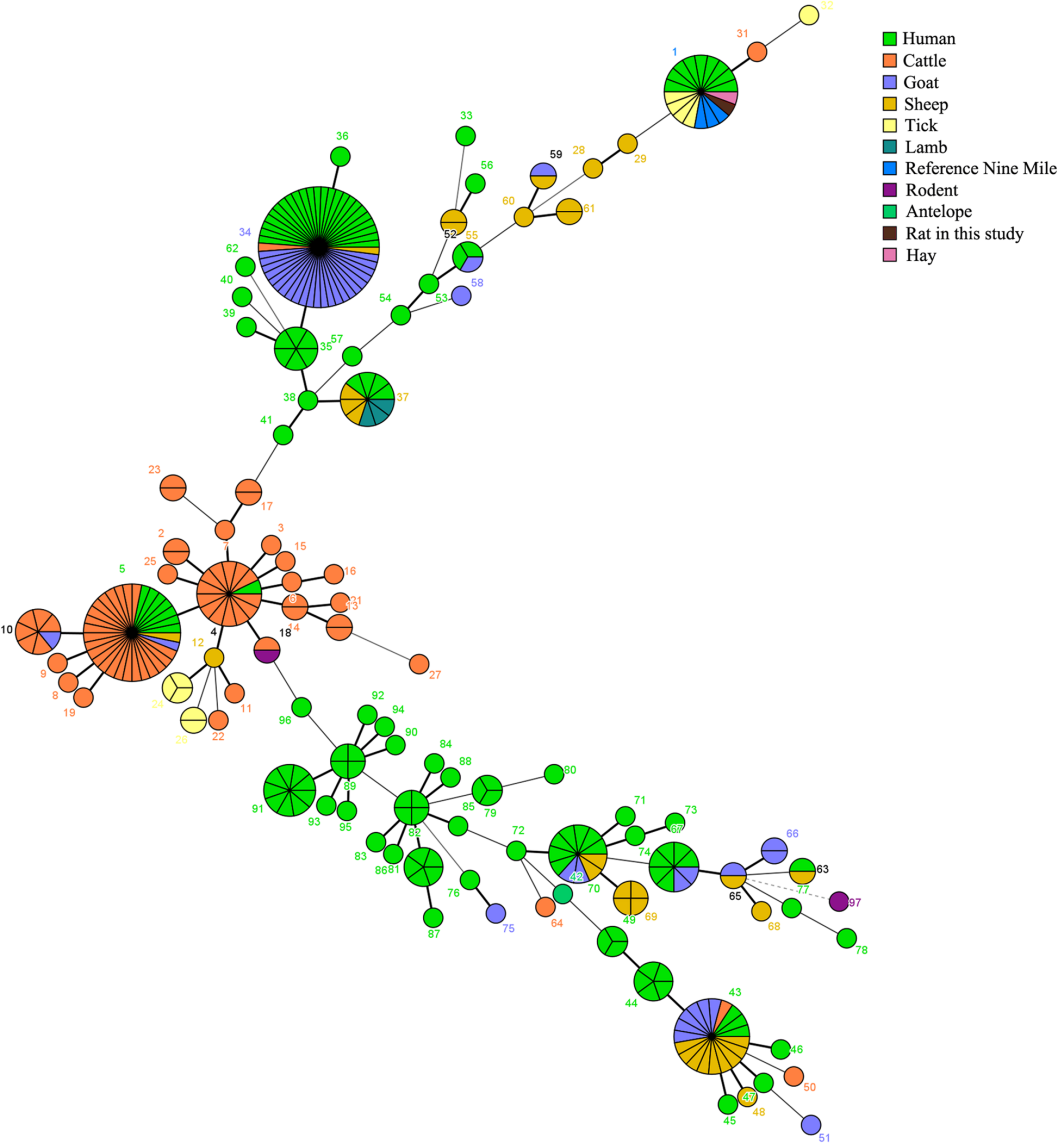
Minimum spanning tree of *Coxiella burnetii* strains based on the results of MLVA-6 analysis. All data of selected samples are available in the MLVA-6 database (http://mlva.i2bc.paris-saclay.fr/mlvav4/genotyping/). The minimum spanning tree provides information on the proportion of hosts of each identified genotype (see color index). Each circle represents a unique genotype, and the size of the pie charts represents the number of isolates of the corresponding genotype

The strains in the 8 liver samples positive for Cb were characterized by the MLVA analysis. The allele codes found in the present study were 9–27-4–6-9–5 for loci ms23-ms24-ms27-ms28-ms33-ms34, and belonged to MLVA genotype 1, suggesting that all Cb strains identified in this study belong to the same genotype. MLVA genotype 1 has already been detected in ticks in the USA, and identified in Q fever patients in the USA, Canada, and France (Fig. 3). A minimum spanning tree based on host origin of the MLVA analysis showed that the Cb strains detected in the present study were clustered with the previously described genotypes found primarily in ticks or Q–fever patients in different regions of the globe (Fig. 4).

## Discussion

In China, Q fever was initially reported in 1950 and the Cb strain was first isolated from a chronic Q fever patient in Chongqing city in 1962 [22]. There have been dozens of studies focused on Q fever and the Cb strains detected and/or isolated from patients [22, 23], mammals [24], wild animals [20] and ticks [25]. However, very little information is available regarding the diversity of Cb strains in China. This study describes the genetic diversity of Cb strains detected in wild rats in Yunnan Province of China using MST and MLVA genotyping, which may be great value in tracking the disease [18, 19] and deciphering its potential zoonotic role in Yunnan.

To date, 74 MST genotypes of Cb have been recorded worldwide in the *Coxiella* MST database (https://ifr48.timone.univ-mrs.fr/mst/coxiella_burnetii/). In the present study, 2 samples (4 and 44) were recognized as MST genotype 16. Interestingly, the allele codes of Cox20 and/or Cox 37 from samples 5, 16, 20, 21 and 22 were designated as novel ones since no correspondence was found among the loci repeats profiles detected herein and those currently recorded in the *Coxiella* MST database, suggesting novel Cb genotypes circulating in China. Up to now the information regarding Cb diversity in China has been found only for strains from hedgehogs samples [20], describing two novel MST genotypes that are different from those typed in the present study.

Using phylogenetic analysis, the two novel MST genotypes in the present study were placed in a clade with MST genotypes 16 and 17, closer to genotype 16. MST genotype 16 is most often associated with Q fever patients and is occasionally recorded in cows or ticks, and it has a widespread distribution in the USA, Japan, Europe (Romania, France, Italy, Germany, Slovakia, Poland), and Central Africa [26]. MST genotype 17 has only been detected in Q fever patients in France according to the MST database. The presence of novel MST genotypes in the present study reinforces this cartographic feature of the genetic diversity based on MST typing of Cb strains.

Different genotyping methods of Cb strains seem to agree with each other [27, 28]. In addition to MST genotyping, MLVA genotyping was performed in the present study. The strains from the rats in Yunnan were recognized as genotype 1 using 6 MLVA loci (ms21, ms22, ms23, ms28, ms33, and ms34) (Fig. 3), and this genotype was mainly found in strains both from Q fever patients and ticks according to the database of MLVABank (http://mlva.u-psud.fr/mlvav4/genotyping/).

A minimum spanning tree allowing the observation of possible host-adapted lineages was drawn (Fig. 4). The obtained strains recognized as MLVA genotype 1 in the present study were clustered mainly with the strains from Q fever patients in France, Canada and the USA, suggesting that these strains from wild rats in Yunnan Province were linked in proximity to the Cb strains from ticks and patients in different regions of the world. This result seems to agree with the result of the MST genotype described above. In our previous study, Cb strains from ticks in Yunnan Province were also typed as MLVA genotype 1 [21], suggesting a wider distribution of this genotype in wildlife in this area.

Although novel MST genotypes of Cb in wild rats were identified in this study, it still has some limitations. The samples were collected only from rats from two locations in Yunnan Province and the number of tested samples was limited. More samples should be collected for analysis in future studies. Moreover, since cases of Q fever have been reported in Yunnan Province [22], there are no Cb genotyping data from patients with Q fever. More studies on the genotyping and pathogenesis of the strains isolated from patients, livestock, wild animals and ticks are necessary to better understand their roles in the epidemiology of Q fever in the province.

## Conclusions

Two novel MST types were identified in wild rats collected from Yunnan Province, and the Cb genotypic diversity in wild rats was reported for the first time in China. Further studies are needed to explore the presence of more genotypes and to associate the genotypes circulating in the wildlife-livestock interaction with those causing human disease to further expand on the epidemiological aspects of the pathogen.

## Methods

### Ethics

All animal care and experimental procedures were in accordance with institutional policies to ensure the highest level of animal health and well-being and were approved by the Institutional Animal Care and Use Committee (IACUC) of the Academy of Military Medical Science (AMMS, Beijing, China) (approval number: IACUC-DWZX-2021–066).

### Sample collection

An investigation was conducted in August 2020, and rats were collected from wild fields in Mengla County and Menglian County in Yunnan Province, China. Rat species were identified based on morphological characterization and by molecular biology methods based on the sequences of the species-specific mitochondrial cytochrome c oxidase I (*COI*) gene as previously described [29]. Rats were anesthetized with pentobarbital sodium (100 mg/kg body weight) and euthanized via cervical dislocation to collect the liver.

### DNA extraction

Livers from all the mice were individually homogenized in 300 μL of phosphate buffered saline (PBS), and then the homogenate was subjected to DNA extraction using a QIAamp® Fast DNA Tissue Kit (Qiagen, Dusseldorf, Germany) following the procedure described before [21]. The extracted genomic DNA was stored at -20 °C.

### Polymerase Chain Reaction (PCR)

Nested PCR targeting the *16S rRNA* gene of Cb was used as described previously [30]. PCR amplifications were carried out in a 50 μL reaction mixture containing 1 × PrimeSTAR® HS (Premix) (TaKaRa, Beijing, China). The PCR products were electrophoresed on a 1.5% agarose gel and visualized under UV light. The positive amplicons were sequenced by TSINGKE Biological Technology (Beijing, China).

Samples positive for Cb were then tested by a quantitative PCR (qPCR) targeting the *com1*gene of Cb as described previously [31].

### Multispacer Sequence Typing (MST)

MST was performed in PCR targeting 10 spacers that exhibited the highest variability, including Cox2, Cox5, Cox18, Cox20, Cox22, Cox37, Cox51, Cox56, Cox57 and Cox61 [2, 18]. Consensus sequences of each Cox spacer were blasted and compared with the sequences in the web-based MST database (https://ifr48.timone.univ-mrs.fr/mst/coxiella_burnetii/). Then the combination of alleles coded of all loci was used to assign the MST genotype. The MST genotypes identified in the present study were compared to genotypes included in the MST database and a phylogenetic analysis was performed using the MEGA X software.

### Multilocus Variable Number Tandem Repeats Analysis (MLVA)

MLVA was performed in PCR targeting six highly variable loci, including ms23, ms24, ms27, ms28, ms33, and ms34 [19]. The forward and reverse primer sequences and PCR conditions were applied as described previously [32–34]. The Nine Mile strain of Cb (RSA493) which was considered 9–27-4–6-9–5 for loci ms23-ms24-ms27-ms28-ms33-ms34 was used as the reference. The MLVA pattern of the strains in the present study was identified using the MLVABank database (http://mlva.u-psud.fr/mlvav4/genotyping/). Clustering of the obtained MLVA profiles was performed with Bionumerics v.7.6 software (Applied Maths, Belgium).

## Data Availability

All data generated or analyzed during current study are available in the GenBank (https://www.ncbi.nlm.nih.gov/WebSub/?form=history&tool=genbank, BankIt2545710).

## Abbreviations

Cb: *Coxiella burnetii*
RFLP-PFGE: Restriction fragment length polymorphism and pulsed-field gel electrophoresis
SNP: Single nucleotide polymorphism
MST: Multispacer sequence typing
MLVA: Multilocus variable number tandem repeats analysis
*COI*: Mitochondrial cytochrome c oxidase I
PBS: Phosphate buffer saline
PCR: Polymerase Chain Reaction
qPCR: Quantitative PCR
